# Silicone oil opacification without emulsification: a rare complication following pars plana vitrectomy

**DOI:** 10.22336/rjo.2025.66

**Published:** 2025

**Authors:** Ashish Markan, Ramesh Kumar Sharma, Parshant Singla, Ramandeep Singh

**Affiliations:** 1 Post Graduate Institute of Medical Education and Research, Chandigarh, India; 2 Sophisticated Analytical Instrumentation Facility, Panjab University, Chandigarh, India

**Keywords:** silicone oil opacification, PPV, retinal detachment, FTIR, TGA, mass spectrometry analysis, BCVA = Best-corrected visual acuity, FTIR = Fourier Transform Infrared Spectroscopy, IOP = Intraocular pressure, LE = Left eye, m/z = Mass-to-charge ratio, PDMS = Polydimethyl siloxane, PPV = Pars plana vitrectomy, SO = Silicone oil, TGA = Thermogravimetric analysis

## Abstract

We report a case of a young patient in his mid-20s who underwent retinal detachment surgery four years before. The patient was lost to follow-up for 4 years. At present, the patient presented to our centre with loss of vision in the operated eye. Examination revealed an oil-filled globe with no signs of oil emulsification. Instead, the oil had lost its transparency and had become opacified. The oil sample was removed and subjected to Fourier Transform Infrared Spectroscopy (FTIR), Thermogravimetric analysis (TGA), and mass spectrometry analysis to ascertain the cause of oil opacification.

## Introduction

Oil opacification is a rarely described complication following silicone oil injection in the eye [1,2]. Oil opacification differs from oil emulsification, referring to the loss of transparency of silicon oil without any breakdown into smaller particles. We hereby report a case of oil opacification in a young patient who underwent retinal detachment surgery 4 years before. Additionally, we described the results of Fourier Transform Infrared Spectroscopy (FTIR), Thermogravimetric analysis (TGA), and mass spectrometry analysis carried out to ascertain the cause of oil opacification.

## Case report

**Fig. 1:**
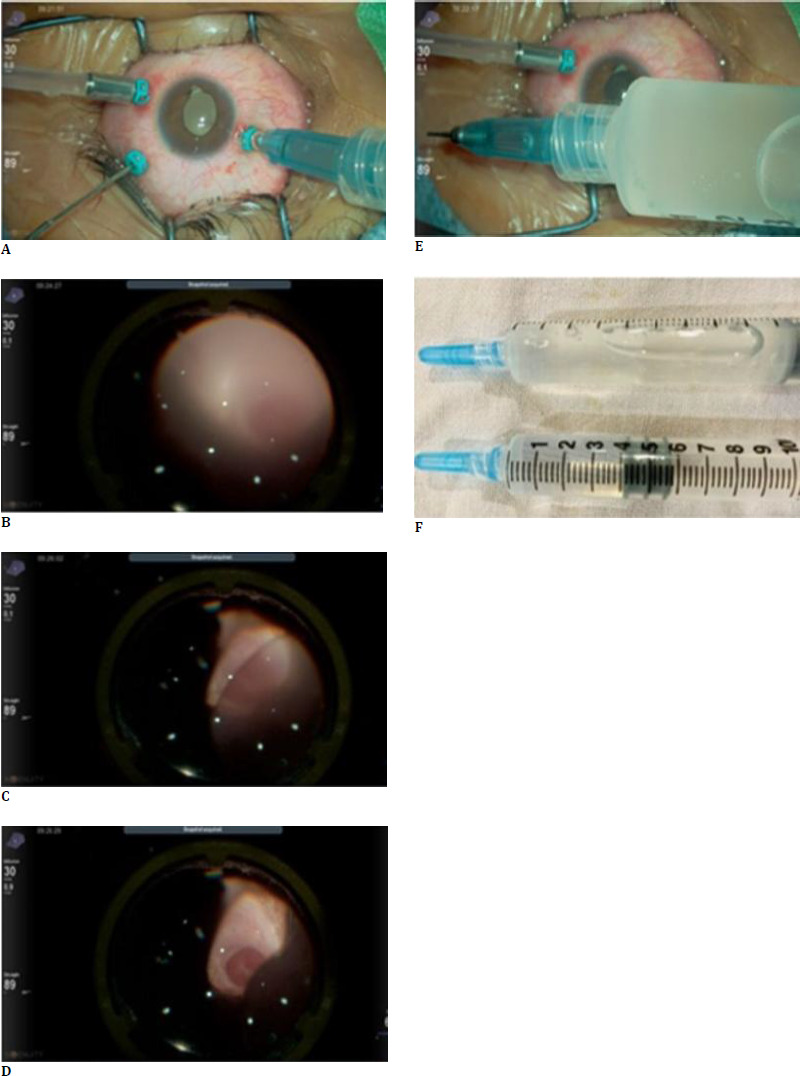
Intraoperative photo showing poor fundus glow and lack of visualization of retinal details because of opacified oil (**A, B**). As the oil is removed, the fundus details become visible. The retina was attached, and a choroidal coloboma was present (**C, D**). The extracted oil was opaque and had lost its transparency (**E**). A comparison of opaque oil with normal silicone oil shows a lack of visualization through the opacified oil as compared to normal silicone oil (**F**)

A young male in his mid-20s presented to our centre with a diminution of vision in the left eye (LE) for the last 2 years. He had a history of type 1 choroidal coloboma with retinal detachment for which he underwent LE pars plana vitrectomy (PPV) with silicon oil (SO) tamponade under local anaesthesia 4 years back. He was lost to follow-up since then. The best-corrected visual acuity (BCVA) was hand motion, close to the face, and the intraocular pressure (IOP) was 18 mmHg. There were no signs of SO emulsification at presentation. Anterior segment examination revealed surgical aphakia and a poor fundus glow. Fundus examination revealed dense oil opacification, no signs of oil emulsification, and no view of retinal details (**Fig. 1A, B**).

The patient was planned for SO removal after written informed consent. The removed oil was sent for chemical analysis to determine the cause of oil opacification (**Fig. 1C-F**). Additionally, a sample of normal SO and an emulsified sample of SO were also sent as controls. The normal SO, emulsified SO, and opacified SO were labelled as samples 1, 2, and 3, respectively.

## Investigations

### Fourier Transform Infrared Spectroscopy (FTIR) analysis

**Fig. 2:**
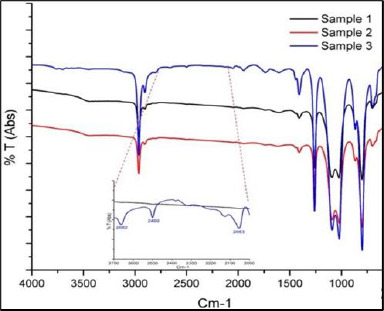
FTIR analysis of normal silicone oil (sample 1), emulsified silicone oil (sample 2), and opacified silicone oil (sample 3).

Fourier Transform Infrared Spectroscopy (FTIR) analysis was performed in the range of 4000 cm-1 to 450 cm-1 (Fig. 2) using a Perkin Elmer Spectrum-400 system (USA). All three samples showed absorption peaks at 2963 cm-1 (corresponding to C-H stretching in the methyl group) and 1261 cm-1 (corresponding to Si-CH3). These peaks represent the typical spectrum of silicone oil composition, specifically polydimethyl siloxane (PDMS) with no impurities. However, sample 3 showed some additional peaks at 3758, 3702, 2662, 2499, and 2053 cm-1 (**Fig. 2**). The peaks at 3758 and 3702 cm-1 indicated the presence of the Si-OH group in the oil sample [3]. Peaks at 2662, 2499, and 2053 cm-1 indicated the presence of NH-H, C≡N, or C≡C groups, respectively, in the removed oil sample. The presence of the Si-OH peak suggested that it might be due to the presence of water, which had subsequently formed a bond with Si from SO. Thus, FTIR analysis did reveal the presence of some impurities in the removed oil.

### Thermogravimetric analysis (TGA)

**Fig. 3:**
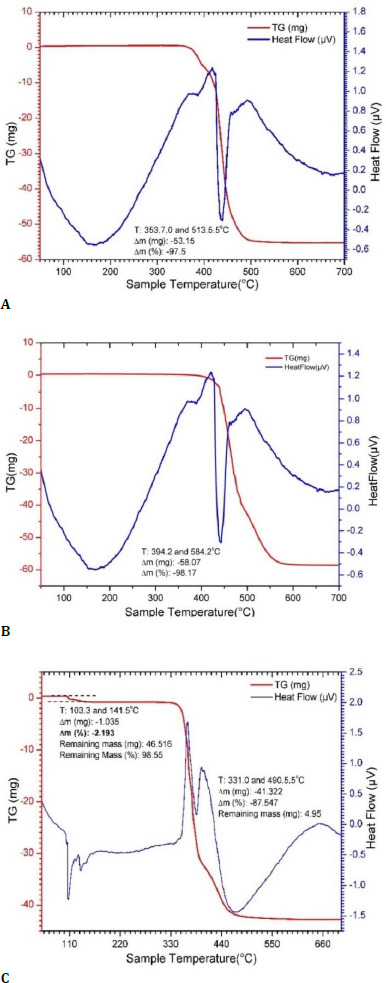
Thermogravimetric analysis of normal silicone oil (**A**), emulsified silicone oil (**B**), and opacified silicone oil (**C**)

Thermogravimetric analysis was performed using a Setaram SETSYS fully computer-controlled instrument (France) (**Fig. 3A-C**). Thermogravimetric analysis (TGA) is a method used to study the weight loss of a sample as it is heated under controlled temperature, providing information about its thermal stability and composition of the material [4]. The temperature was scanned from room temperature to 700°C at a rate of 10°C increase. The samples were taken in the range of 50 to 60 mg. TGA analysis showed weight loss in the temperature range of 353°C and 513°C in all three samples. This weight loss was primarily due to evaporation of SO, which was an expected behavior of SO. However, in sample 3, a minor weight loss was also observed between 103°C and 141°C (**Fig. 3C**). The weight loss was around 2%, which was significant. This weight loss was attributed to the loss of water. This observation supported the presence of the Si-OH bond in the FTIR analysis.

### Mass spectrometry analysis

**Fig. 4:**
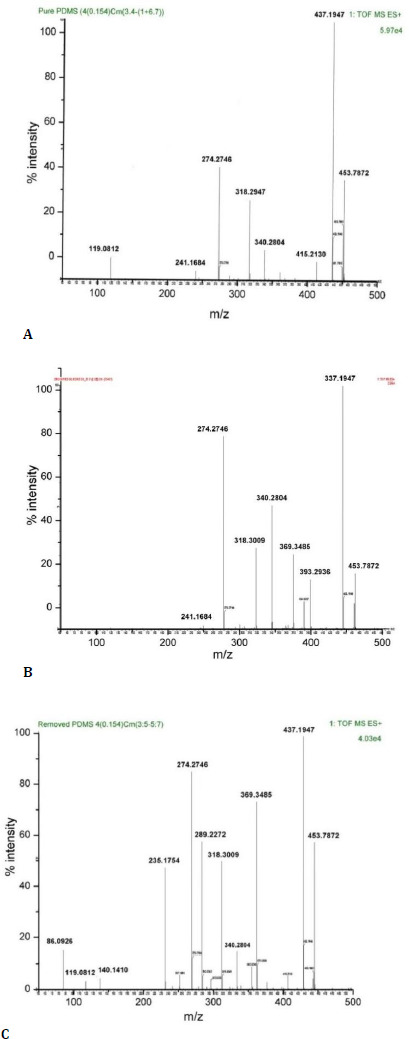
Mass spectrometry analysis of normal silicone oil (**A**), emulsified silicone oil (**B**), and opacified silicone oil (**C**)

Samples were further analyzed using an advanced high-resolution mass spectrometer (Synapt-XS, Waters Corporation, Milford, USA) (**Fig. 4A-C**) to ascertain the reason for the extra functional groups found on FTIR analysis in sample 3. All three samples showed identical spectra with m/z values at 453, 437, 415, 340, 318, 274, and 119, indicating the nature of the basic SO. But interestingly, sample 3 also showed four extra peaks at m/z 289, 235, 140, and 86 (**Fig. 3C**). The exact mass of these peaks came out to be 289.2272, 235.1754, 140.1410, and 86.026 m/z in mass spectrometry, which represents the mass-to-charge ratio of an ion [5]. The ions are separated based on mass-to-charge ratio. This ratio is used for the identification and characterization of ions. Mass spectra display ion abundance relative to their m/z values.

The M+1 peak at 235.1754 and 86.026 indicates the presence of Lidocaine, which has its theoretical value of accurate mass at 234.1732 m/z (C14H22N2O) and its fragment at 85.0891 m/z (C5H11N), whereas M+1 peak at 289.2272 and 140.1410 indicate the presence of Bupivacaine, which has its theoretical value of accurate mass at 288.2201 (C18H28N2O) and its fragment at 139.1361 (C9H17N). The precise mass study confirmed the presence of Lidocaine and Bupivacaine in the oil removed from the eye.

In Electrospray Ionization (ESI) mass spectrometry, M+1 or M+H refers to the ion formed by adding a proton (H+) to the molecule of interest (M) during the ionization process. M+H ions are commonly observed in ESI positive ionization mode and provide valuable information about the molecular weight and structure of the compound being analyzed.

## Discussion

Opacification of SO is an infrequent complication during the post-operative period [1,2]. Opacification of the oil is different from oil emulsification. Emulsification is the process by which water is dispersed into oil in the form of small droplets. Water droplets can remain in an oil layer in a stable form, and the properties of the emulsified oil are significantly different from those of the original oil [6,7]. It results from shearing forces between two media, causing droplets to be pinched off into another medium because of surface tension. Several factors, including low viscosity, the presence of biological emulsifiers such as blood, fibrin, and serum, contamination from surgical instruments, and oil underfill, are known to promote silicone oil emulsification in the postoperative period [8].

On the other hand, opacification of SO is rarely reported. Opacification refers to the loss of transparency of SO without any signs of oil emulsification. Chandra et al. reported a case of oil opacification in a young child, who was lost to follow-up and presented after a year [1]. The removed silicon oil was opaque and white in color. The authors were unable to determine the cause of oil opacification and concluded that oil can lose its transparency upon prolonged retention, suggesting that it should be removed promptly. Zheng et al. showed an early opacification of SO in 13 cases undergoing vitreoretinal surgery. Physiochemical analysis revealed that opacified oil was more heat-stable and lacked a stabilizing agent against coloration. The oil used in all these cases was traced to a single production lot. Additionally, the use of intraoperative adjuncts, such as perfluorocarbon liquid, can cause the formation of an opaque fluid due to the microdispersion of PFCL liquid into the SO [9]. The above reports highlight the change in physicochemical properties of the SO composition, resulting in it losing its transparency.

FTIR and mass spectrometry analysis of the opacified oil in our study revealed the presence of additional Si-OH bonds and impurities, such as lidocaine and bupivacaine, which were absent in the control groups. The opacification can be attributed to the presence of Si-OH bonds. These additional bonds are likely formed due to prolonged contact of SO with the aqueous fluid.

Though the role of lidocaine and bupivacaine in causing opacification cannot be ascertained, their presence in the oil sample cannot be overlooked. A mixture of lidocaine and bupivacaine is the most commonly used preparation for ophthalmic anesthetic blocks [10]. At our centre, we prefer retrobulbar block utilizing a mixture of lidocaine and bupivacaine in the inferior quadrant. This patient also underwent primary surgery under local anaesthesia using a retrobulbar block. Drug deposition in the retrobulbar space is in proximity to the scleral surface. Studies have shown direct penetration routes (transscleral via periocular drug deposition) to provide superior drug delivery into the vitreous cavity, when compared with oral or topical routes. We believe that trans-scleral diffusion of the drug would have caused the anaesthetic mixture to gain access into the eye in our case [11].

Transscleral drug diffusion from the periocular route involves various membrane barriers, including the choroid, Bruch’s membrane, retinal pigment epithelium, and neurosensory retina [12,13]. Apart from this, clearance through choroidal circulation and binding to melanin pigments can limit drug delivery into the vitreous cavity [14,15]. The absence of choroid and retinal tissue in a large inferior coloboma may have accelerated the process of drug diffusion through the transscleral route in our case. This probably explains the presence of a significant amount of lidocaine and bupivacaine in the oil sample removed. Whether the presence of lidocaine and bupivacaine per se caused the opacification of clear oil, altered the chemical bonds, or accelerated the process of Si-OH could not be ascertained.

## Conclusion

In conclusion, we reported a rare case of oil opacification following retinal detachment surgery in the postoperative period. Long-term retention can result in the formation of Si-OH bonds, causing the oil to lose its transparency. This can be visually disabling to the patient and requires oil removal.
